# Combined Analysis of Metabolome and Transcriptome Reveals *Bauhinia variegata*-Specific Floral Scent Profile and Key Aroma Components

**DOI:** 10.3390/metabo16090611

**Published:** 2026-08-26

**Authors:** Zhijiao Song, Guixiang Li, Wenhua Chen, Qing Liu, Yantong Teng

**Affiliations:** 1 College of Resources and Environmental Sciences, Baoshan University, Baoshan 678000, China; zhijiaosong@bsc.edu.cn (Z.S.);; 2 Yunnan Academy of Forestry and Grassland, Kunming 650201, China

**Keywords:** *Bauhinia variegata*, floral volatile organic compounds (VOCs), floral scent profile, metabolome, transcriptome

## Abstract

Background: *Bauhinia* *variegata* is a plant with considerable application potential owing to its combined ornamental, edible, aromatic, and medicinal values. However, research on this species remains limited and superficial both domestically and internationally, and systematic investigation of floral volatile organic compounds (VOCs) is still lacking. Methods: Through integrated metabolome and transcriptome analyses. Results: This study first comprehensively characterizes the VOC composition, floral scent profile, key aroma components, and the molecular mechanisms underlying VOC variation during anthesis in floral buds and flowers of *B. variegata*. A total of 1214 volatile compounds were identified across buds and flowers, including 239 odor-active compounds and 35 differential odor-active compounds. Flavor statistics revealed that the floral scent profile of *B. variegata* is dominated by fruity, sweet, floral, green, woody, herbal, citrus, phenol, fresh, and spicy notes. Compared to floral buds, most differential odor-active compounds were markedly upregulated in flowers, including key floral aroma constituents such as phenylacetaldehyde, rose oxide, (Z)-β-ocimene, 2-methylbenzaldehyde, and melon heptenal. Conversely, (R)-(+)-citronellal, which possesses defensive functions, and the bitter-tasting compound 1-methyl-4-nitro-benzene were significantly downregulated in flowers, reflecting a shift from a defense-oriented mode at the bud stage to an attraction-oriented mode at anthesis. Upregulation of phenylalanine/histidine ammonia-lyase, acyl-CoA synthetase, and squalene synthetase genes and downregulation of copper amine oxidase, O-methyltransferase, and aldo–keto reductase genes synergistically promoted accumulation of floral aroma compounds such as phenylacetaldehyde and facilitated the floral transition. Conclusions: This study provides important data support for understanding the ecological interactions between *B. variegata* floral scent and its pollinators, as well as the molecular mechanisms governing floral scent formation. Furthermore, it contributes to the application of *B. variegata* in landscaping, edible flower utilization, and fragrance development.

## 1. Introduction

*Bauhinia* belongs to the family Fabaceae (Leguminosae) and is named for its characteristic bilobed leaves resembling the hooves of a sheep or camel. The genus *Bauhinia* is highly diverse in species richness and morphological variation, comprising trees, shrubs, and lianas. It encompasses over 300 known species, with commonly reported species including *B. variegata*, *B. blakeana*, *B. purpurea*, *B. championii*, *B. galpinii*, *B. integrifolia*, *B. kockiana*, *B. malabarica*, *B. phoenicea*, *B. racemosa*, *B. retusa*, *B. scandens*, *B. tomentosa*, *B. acuminata*, and *B. vahlii*. *Bauhinia* species are predominantly distributed across tropical and subtropical regions, including Asia, South America, and Africa. Within Asia, *Bauhinia* is primarily found in countries such as India, China, Thailand, Malaysia, Vietnam, Myanmar, and the Himalayan region [1].

Flowers, with their appealing colors, fragrances, flavors, and rich nutritional profiles, have a long-standing tradition of culinary use among people worldwide, including in Asia, ancient Greece and Rome, Medieval Europe, France, Victorian England, and the Middle East. Edible flowers include rose, marigold, gerbera, chrysanthemum, jasmine, tuberose, gladiolus, carnations, *B. variegata*, *Bombax ceiba*, and *Sesbania grandiflora*, among which *Bauhinia* is included [2,3,4]. Beyond its ornamental value and traditional dietary uses, the genus *Bauhinia* also possesses significant medicinal properties. Various tissues of *Bauhinia* species, including *B. variegata*, have been reported to exhibit antimicrobial, anti-inflammatory, antioxidant, nephroprotective, hepatoprotective, anticancer, and antidiabetic activities and are widely utilized in traditional folk medicine in countries such as India [5,6,7,8,9].

In China, the genus *Bauhinia* is mainly distributed in the southern regions, including Hong Kong, Taiwan, Yunnan, Guangdong, Guangxi, and Hainan. *Bauhinia blakeana* is the city flower of Hong Kong and is extensively used in urban landscaping across various regions due to its high ornamental value, characterized by profuse flowering, pleasant fragrance, and a long blooming period. The tradition of consuming flowers in China is as ancient as its history and culture, particularly in Yunnan Province, where flowers of *Bauhinia variegata*, *Buddleja officinalis*, *Gmelina arborea*, *Smilax zeylanica*, and *Musa* spp. have rich records of culinary use [10].

Floral volatile organic compounds (VOCs) play critical roles in attracting pollinators and seed dispersers, defending against herbivores, protecting against plant pathogens, mediating plant–plant communication, and shielding plants from abiotic stresses. Additionally, they hold substantial economic importance in the perfume, cosmetics, food, beverage, and pharmaceutical industries [11,12,13]. Different plant species exhibit species-specific floral volatile profiles, which are crucial for their reproduction and environmental adaptation [14].

*Bauhinia* possesses considerable application potential in ornamental horticulture, edible flower production, fragrance development, and medicine. However, research on this genus, both domestically and internationally, remains relatively superficial, thereby limiting its further application and promotion. To date, a systematic investigation of the volatile organic compounds emitted from the flowers of *B. variegata* is still lacking. This study provides, for the first time, a comprehensive characterization of the volatile organic compound composition, floral scent profile, and key aroma components of *B. variegata* floral buds and flowers. This work provides important data support for a deeper understanding of the ecological interactions between *B. variegata* floral scent and its pollinators, as well as the molecular mechanisms governing floral scent formation. Concurrently, it contributes to the application of *B. variegata* in landscaping, edible flower utilization, and fragrance development.

## 2. Materials and Methods

### 2.1. Plant Materials

*B. variegata* floral buds and opened flowers were collected from the campus of Baoshan University, Yunnan, China, between 9:00 and 10:00 AM on sunny days to minimize diurnal variation in volatile emission. Samples were immediately frozen in liquid nitrogen and stored at −80 °C. Three biological replicates were prepared per stage, each consisting of approximately 5 g of floral tissue pooled from multiple individuals. All samples were ground to a fine powder in liquid nitrogen using a pre-chilled mortar and pestle prior to extraction.

### 2.2. Volatile Organic Compounds Extraction and GC–MS Analysis

Volatile organic compounds (VOCs) were extracted using headspace solid-phase microextraction (HS-SPME) [15,16]. Approximately 500 mg of frozen sample powder was transferred into a 20 mL headspace vial containing 5 mL of saturated NaCl solution and 20 μL of internal standard (2-octanol, 10 μg/mL in methanol). The vial was sealed with a crimp-top cap. A 120 μm DVB/CAR/PDMS SPME Arrow fiber (Agilent Technologies, Santa Clara, CA, USA) was conditioned at 250 °C for 5 min and then exposed to the sample headspace at 60 °C for 15 min after 5 min of equilibration. Desorption was performed at 250 °C for 5 min in splitless mode.

GC–MS analysis was conducted on an Agilent 8890 GC coupled to a 7000E MS with a DB-5MS capillary column (30 m × 0.25 mm i.d., 0.25 μm film thickness). Helium carrier gas flow was 1.2 mL/min. The oven program was 40 °C for 3.5 min, ramped up to 100 °C at 10 °C/min, 180 °C at 7 °C/min, and 280 °C at 25 °C/min, and held for 5 min. Injector temperature was 250 °C. The MS was operated in EI mode at 70 eV; quadrupole, ion source, and transfer line temperatures were 150 °C, 230 °C, and 280 °C, respectively [17]. Full scan data (*m*/*z* 30–550) were acquired for compound identification, and selected ion monitoring (SIM) was used for quantification of target compounds. Identification was achieved by matching retention times and mass spectra with the Metware in-house database and the NIST library and by comparison of retention indices calculated using n-alkanes (C_8_–C_40_). Relative quantification of each volatile compound was performed using the internal standard method. The relative content (Ci, μg/g fresh weight) was calculated based on the peak area ratio of each compound to the internal standard (2-octanol), assuming a response factor of 1.0.

### 2.3. Relative Odor Activity Value Calculation

Odor thresholds and descriptors were retrieved from public databases, namely The Good Scents Company (Oak Creek, WI, USA) (http://www.thegoodscentscompany.com), Flavornet (http://www.flavornet.org), The LRI and Odour Database (https://www.odour.org.uk), and The Food Database (https://foodb.ca), as well as from published literature [18,19,20]. All URLs were accessed on 11 August 2025. The relative odor activity value (rOAV) was calculated as

$$rOAV_{i}=\frac{C_{i}}{T_{i}}$$

where *C_i_* is the relative content (μg/g FW) of compound “*i*”, and *T_i_* is its odor threshold (μg/g). Compounds with rOAV ≥ 1 were considered odor-active compounds.

### 2.4. Differential VOCs Selection and KEGG Pathway Classification

Differential VOCs between buds and flowers were identified using orthogonal partial least squares discriminant analysis (OPLS-DA) with variable importance in projection (VIP) scores. OPLS-DA was performed using the MetaboAnalystR package (version 1.0.1; www.r-project.org, accessed on 11 August 2025). Data were log_2_-transformed and mean-centered; model validity was assessed by 200-fold permutation. Metabolites with VIP > 1, |log_2_FC| ≥ 1, and *p* < 0.05 (based on Student’s *t*-test) were considered significantly differential. Annotation and pathway classification were performed against the KEGG database (https://www.kegg.jp, accessed on 11 August 2025) using a hypergeometric test with *p* < 0.05 as the significance threshold [21].

### 2.5. RNA Extraction and Transcriptome Sequencing

Total RNA was extracted using a modified CTAB method [22]. Briefly, samples were ground in liquid nitrogen, homogenized in CTAB extraction buffer, and purified by chloroform-isoamyl alcohol extraction followed by lithium chloride precipitation.

RNA quality and concentration were assessed with a Qubit fluorometer (Thermo Fisher Scientific, Waltham, MA, USA) and Qsep400 biofragment analyzer (Bioptic Inc., New Taipei City, Taiwan, China); all samples had RIN ≥ 8.0. cDNA libraries were constructed following the Illumina protocol: mRNA was enriched with Oligo(dT) magnetic beads, fragmented, and reverse-transcribed using random hexamer primers. Second-strand cDNA synthesis incorporated dUTPs for strand-specific libraries. After end repair, dA-tailing, and adapter ligation, 250–350 bp fragments were selected and PCR-enriched. Libraries were quantified by Q-PCR and sequenced on an Illumina NovaSeq 6000 platform, generating 150 bp paired-end reads. Raw reads were filtered with fastp to remove adapter sequences and low-quality reads. Clean reads were de novo assembled using Trinity, and transcripts were clustered with Corset [23,24]. Functional annotation was performed by DIAMOND BLAST (version 2.0.15; E-value < 1 × 10^−5^) against NR, Swiss-Prot, GO, COG/KOG, Trembl, and KEGG databases and by HMMER against Pfam. Gene expression levels were quantified as FPKM using RSEM [25]. Differentially expressed genes were identified using DESeq2 with thresholds of |log_2_fold change| ≥ 1 and FDR < 0.05 [26,27].

### 2.6. Statistical Analysis

Multivariate analyses and visualizations were performed using MetaboAnalystR 4.0 and the Metware Cloud platform (https://cloud.metware.cn, accessed on 11 August 2025). All experiments included three biological replicates, and data are presented as mean ± SD. Metabolite–gene correlation networks were constructed based on Pearson’s correlation coefficients, with |r| ≥ 0.8 and *p* < 0.05 considered significant.

## 3. Results

### 3.1. Identification of VOCs in Floral Buds and Flowers of B. variegata

**Figure 1:**
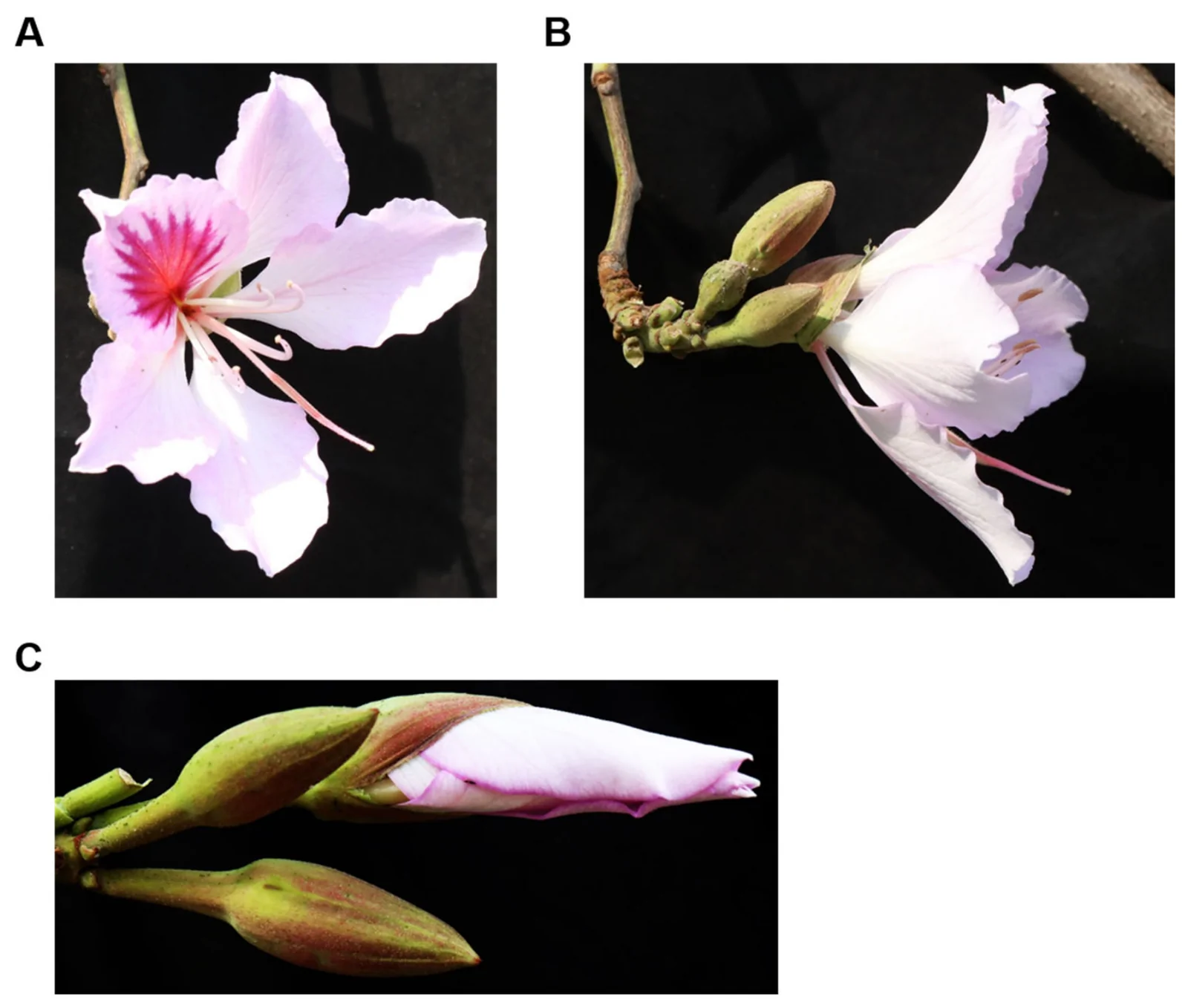
Flower and floral bud phenotypes of *Bauhinia variegata*: (**A**,**B**) flowers, with one of the five petals bearing magenta markings; (**C**) floral bud.

A total of 1214 volatile metabolites were detected in *B. variegata* floral buds (Figure 1C) and flowers (Figure 1A,B) using gas chromatography–mass spectrometry (GC–MS). Detailed information, including compound names, chemical classes, molecular formulas, and CAS registry numbers for these 1214 volatiles, is provided in Table S1. The classification and proportional distribution of these volatiles are illustrated in Table 1. Among the detected volatiles, the following categories were identified: Terpenoids (300), Esters (221), Ketones (119), Heterocyclic Compounds (115), Alcohols (95), Hydrocarbons (74), Phenols (58), Acids (55), Aldehydes (51), Aromatics (45), Amines (25), Ethers (26), Nitrogen Compounds (15), and Sulfur Compounds (12).

### 3.2. Floral Scent Profile of B. variegata and Differential Flavor Analysis Between Floral Buds and Flowers

Odor threshold values and flavor descriptors for the volatile compounds were retrieved from public databases and incorporated into Table S1, which also includes the calculated relative odor activity values. Volatile compounds exhibiting an rOAV ≥ 1 were considered odor-active compounds. In floral buds, 211 odor-active compounds were identified and are listed in descending order in Table S2. In flowers, 231 odor-active compounds were identified and are similarly listed in Table S3. Based on the flavor descriptors of these odor-active compounds in buds and flowers, the floral scent profiles for floral buds and flowers were constructed and are presented in Figure S2 and Figure S3, respectively. Statistical analysis revealed that the floral scent profile of *B. variegata* is predominantly characterized by fruity, sweet, floral, green, woody, herbal, citrus, phenol, fresh, and spicy notes.

Integration of the 211 and 231 odor-active compounds in buds and flowers, respectively, yielded a combined set of 239 odor-active compounds; a Venn diagram illustrating the overlap is provided in Figure S4. Among these 239 compounds, 8 were unique to floral buds, including 2-methoxybenzoic acid (CAS: 579-75-9), (4-methylphenyl) acetate (CAS: 140-39-6), 2,6-dichlorophenol (CAS: 87-65-0), 2,5-dinitrophenol (CAS: 329-71-5), 1-methoxy-3-methylbenzene (CAS: 100-84-5), and (R)-(+)-citronellal (CAS: 2385-77-5), with herbal being a predominant flavor note among these bud-specific volatiles. Conversely, 28 compounds were unique to flowers and are detailed in Table S4.

**Figure 2:**
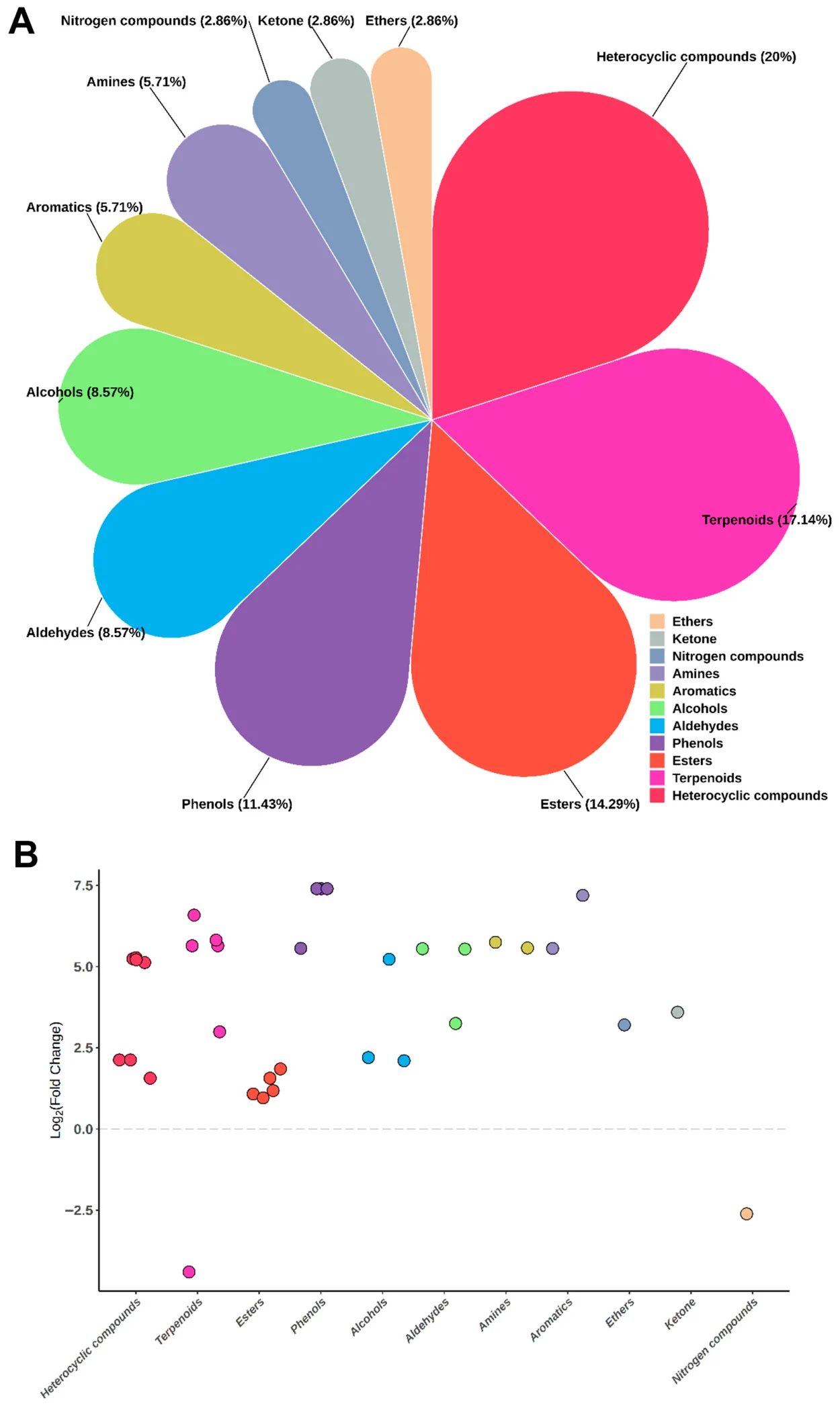
Characterization of differential odor-active compounds: (**A**) pie chart of differential odor-active compounds; (**B**) scatter plot of differential odor-active compounds. The horizontal axis represents volatile compound classes, with differently colored dots denoting distinct compound categories. The vertical axis indicates the log_2_(fold change) in compound content between flowers and floral buds, where values above zero indicate higher content in flowers, values below zero indicate higher content in floral buds, and the further from zero, the greater the difference.

From the combined set of 239 odor-active compounds, 35 were identified as significantly different between floral buds and flowers and are listed in Table S5. The classification and proportion of these 35 differential compounds are shown in Figure 2A and Table 2. The results show that the predominant classes among the differential odor-active compounds are heterocyclic compounds, terpenoids, esters, phenols, aldehydes, and alcohols. Notably, compared to the proportions observed in the total metabolite pool, the proportion of heterocyclic compounds increased markedly. The classification and regulation pattern of these 35 differential odor-active compounds are depicted in Figure 2B. As illustrated, the majority of volatiles were substantially upregulated in flowers relative to floral buds, with only two compounds, (R)-(+)-citronellal and 1-methyl-4-nitro-benzene, exhibiting significant downregulation.

**Figure 3:**
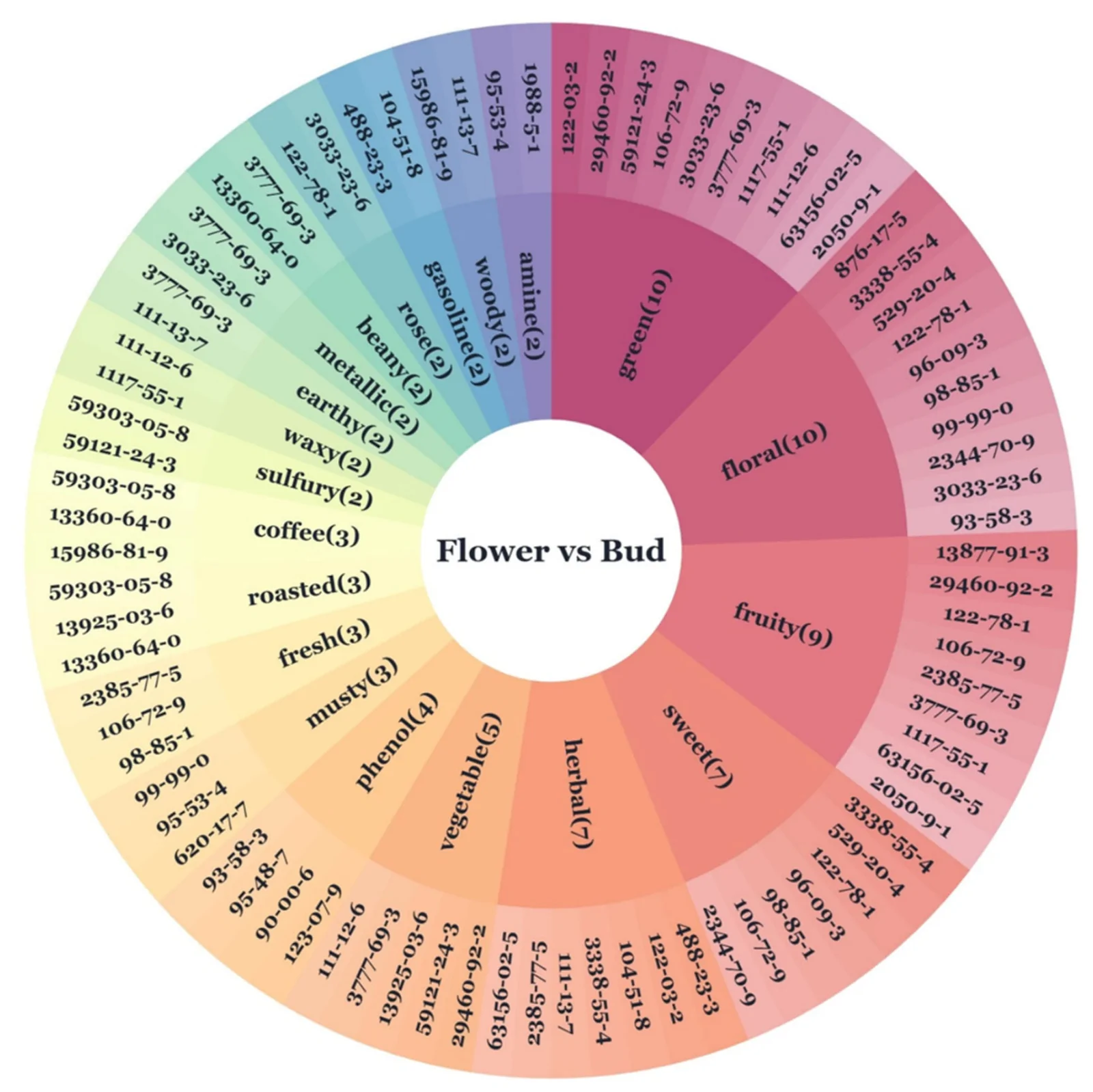
Flavor analysis of differential odor-active compounds between flowers and floral buds. The middle ring represents distinct flavor categories, with different colors indicating different flavor notes. The outer ring displays the CAS numbers of compounds that contribute to the corresponding flavor categories. Within each flavor category, individual metabolites are arranged according to the magnitude of their differential abundance, with darker shading indicating a larger fold change.

Figure 3 presents a differential flavor wheel constructed based on the 35 differential odor-active compounds. Among the downregulated volatiles, 1-methyl-4-nitro-benzene was the sole contributor to a bitter note, while (R)-(+)-citronellal was a key contributor to the herbal note. Compared to floral buds, the primary differential flavor notes in flowers included floral, sweet, fruity, and herbal. Key differential metabolites influencing floral and sweet notes comprised rose oxide (CAS: 876-17-5), (Z)-β-ocimene (CAS: 3338-55-4), phenylacetaldehyde (CAS: 122-78-1), 2-methylbenzaldehyde (CAS: 529-20-4), styrene oxide (CAS: 96-09-3), alpha-methylbenzyl alcohol (CAS: 98-85-1), and 4-phenyl-2-butanol (CAS: 2344-70-9). Differential metabolites impacting fruity notes included β-ocimene (CAS: 13877-91-3), 2-isobutyl pyrazine (CAS: 29460-92-2), and melon heptenal (CAS: 106-72-9). Those affecting herbal notes included 4-isopropylbenzaldehyde (CAS: 122-03-2), 1,2,3,4-tetramethylbenzene (CAS: 488-23-3), butylbenzene (CAS: 104-51-8), and 2-octanone (CAS: 111-13-7).

### 3.3. KEGG Pathway Analysis of Differential Odor-Active Compounds

The 35 identified differential odor-active compounds were subjected to KEGG pathway analysis. Among these, 19 compounds were assigned KEGG metabolite IDs, but only 7 were further annotated with KEGG pathway IDs. Detailed KEGG annotation information is recorded in Table S6. Summary information for these 7 metabolites is presented in Table 3, which includes styrene oxide, 2-methylbenzaldehyde, phenylacetaldehyde, 2-methylphenol, (R)-(+)-citronellal, 4-isopropylbenzaldehyde, and 4-ethylphenol.

The metabolites were associated with pathways including xylene degradation; toluene degradation; styrene degradation; pinene, camphor, and geraniol degradation; phenylalanine metabolism; degradation of aromatic compounds; bisphenol degradation; and biosynthesis of terpenoids and steroids. Detailed information on the pathways and their corresponding metabolites is provided in Table 4.

### 3.4. Expression Analysis of Significantly Differentially Expressed Genes

Analysis of the transcriptomic data identified significantly differentially expressed genes (DEGs) within the KEGG pathways associated with the differential odor-active compounds, with detailed expression data provided in Table S7. A total of 23 significant DEGs were identified in the phenylalanine metabolism pathway, 3 in the biosynthesis of terpenoids and steroids, 8 in styrene degradation, and 6 in the degradation of aromatic compounds. No significant DEGs were detected in the xylene degradation; toluene degradation; pinene, camphor, and geraniol degradation; or bisphenol degradation pathways.

**Figure 4:**
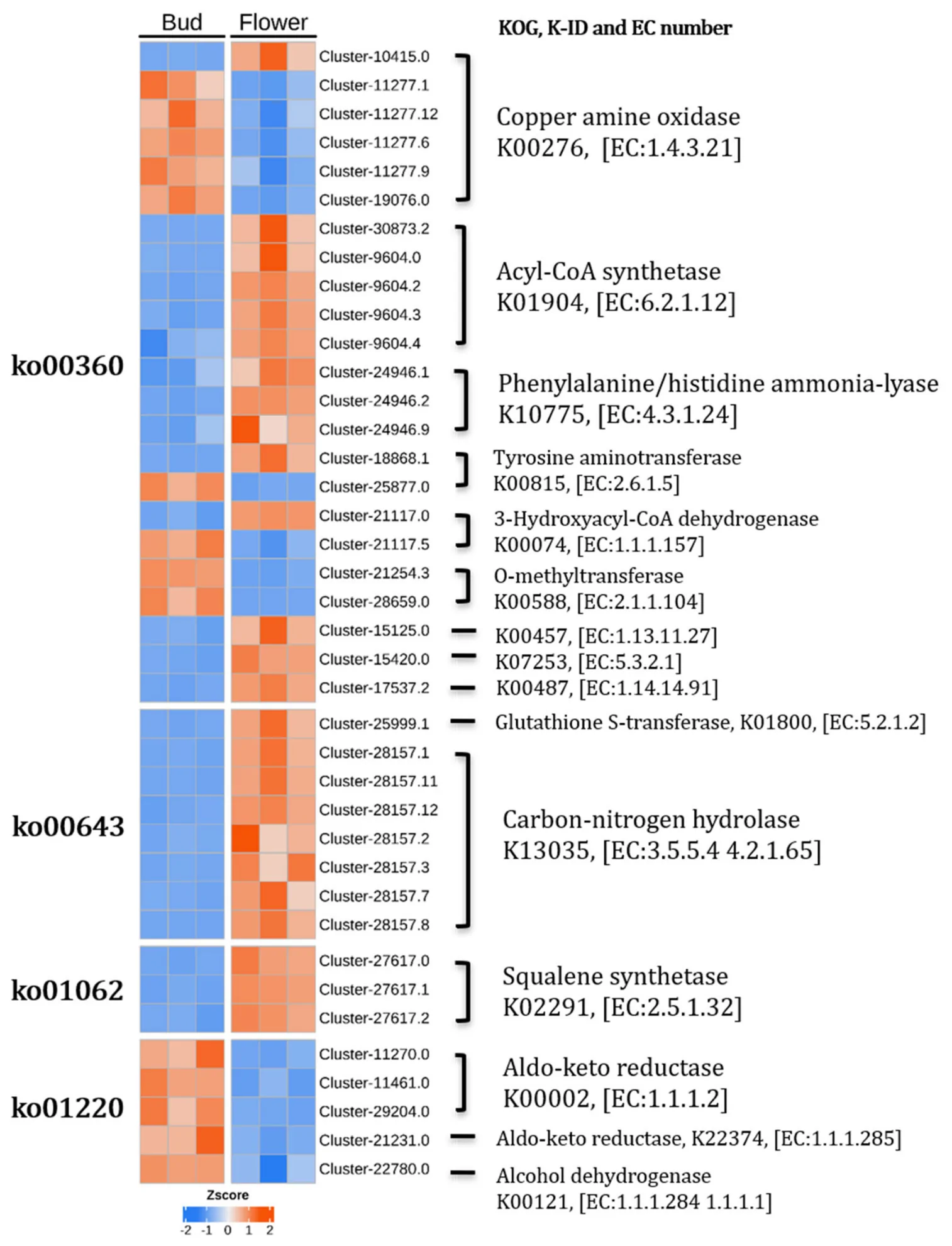
Differentially expressed genes in key pathways. In the heatmap, blue indicates relatively low gene expression, red indicates relatively high expression, and a deeper color indicates a greater difference.

As shown in Figure 4, in the phenylalanine metabolism pathway, six copper amine oxidase genes were identified (five downregulated, one upregulated), along with five acyl-CoA synthetase genes (all upregulated), three phenylalanine/histidine ammonia-lyase genes (all upregulated), two tyrosine aminotransferase genes (one upregulated, one downregulated), two O-methyltransferase genes (both downregulated), two 3-hydroxyacyl-CoA dehydrogenase genes (one upregulated, one downregulated), one upregulated 4-hydroxyphenylpyruvate dioxygenase gene, one upregulated macrophage migration inhibitory factor gene, and one upregulated cytochrome P450 CYP2 subfamily gene. In the biosynthesis of terpenoids and steroids pathway, three squalene synthetase genes were upregulated. In the styrene degradation pathway, seven carbon–nitrogen hydrolase genes were all upregulated, and one glutathione S-transferase gene was upregulated. In the degradation of aromatic compounds pathway, four aldo–keto reductase genes were all downregulated, and one alcohol dehydrogenase gene was downregulated.

### 3.5. Metabolite–Gene Correlation Analysis

**Figure 5:**
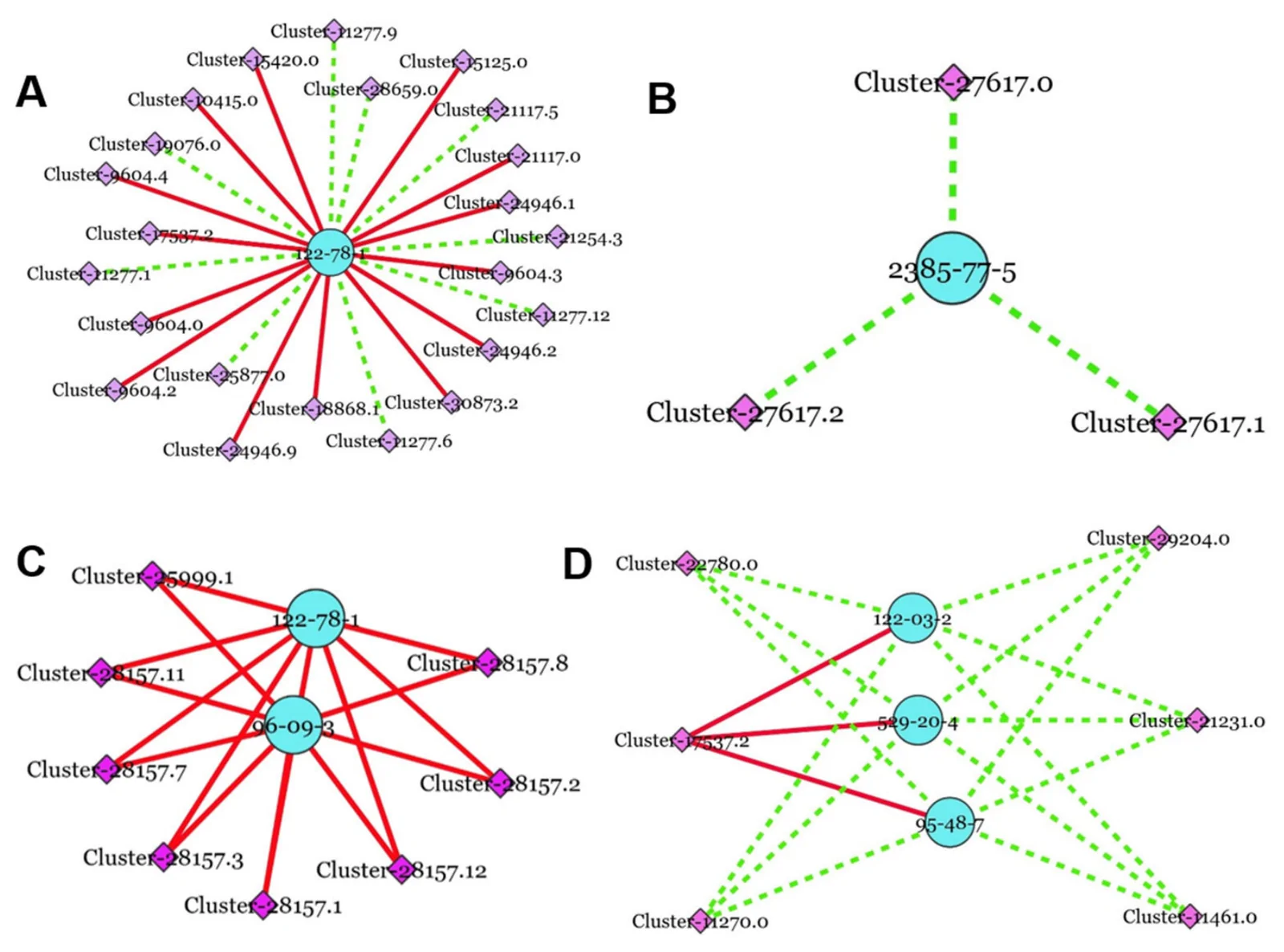
Metabolite–gene correlation analysis: (**A**) Correlation network for KEGG pathway ko00360 (phenylalanine metabolism). (**B**) Correlation network for KEGG pathway ko01062 (biosynthesis of terpenoids and steroids). (**C**) Correlation network for KEGG pathway ko00643 (styrene degradation). (**D**) Correlation network for KEGG pathway ko01220 (degradation of aromatic compounds). In each network, cyan circles represent differential odor-active compounds (metabolites), purple diamonds represent significantly differentially expressed genes, red solid lines indicate positive correlations, and green dashed lines indicate negative correlations.

Correlation networks illustrating the relationships between differential odor-active compounds and significantly differentially expressed genes within the aforementioned four pathways are presented in Figure 5. As shown in Figure 5A, within the phenylalanine metabolism pathway, the upregulated metabolite phenylacetaldehyde (CAS: 122-78-1) exhibited a positive correlation with 14 upregulated genes and a negative correlation with 9 downregulated genes. As depicted in Figure 5B, within the biosynthesis of terpenoids and steroids pathway, the downregulated metabolite (R)-(+)-citronellal (CAS: 2385-77-5) was negatively correlated with three upregulated genes. Figure 5C demonstrates that within the styrene degradation pathway, the two upregulated metabolites, styrene oxide (CAS: 96-09-3) and phenylacetaldehyde, were positively correlated with eight upregulated genes. Finally, Figure 5D shows that the three upregulated metabolites, 4-isopropylbenzaldehyde (CAS: 122-03-2), 2-methylbenzaldehyde (CAS: 529-20-4), and 2-methylphenol (CAS: 95-48-7), were negatively correlated with five downregulated genes and positively correlated with one upregulated gene.

### 3.6. Integrated Analysis of Differential Metabolites and Genes in KEGG Pathways

**Figure 6:**
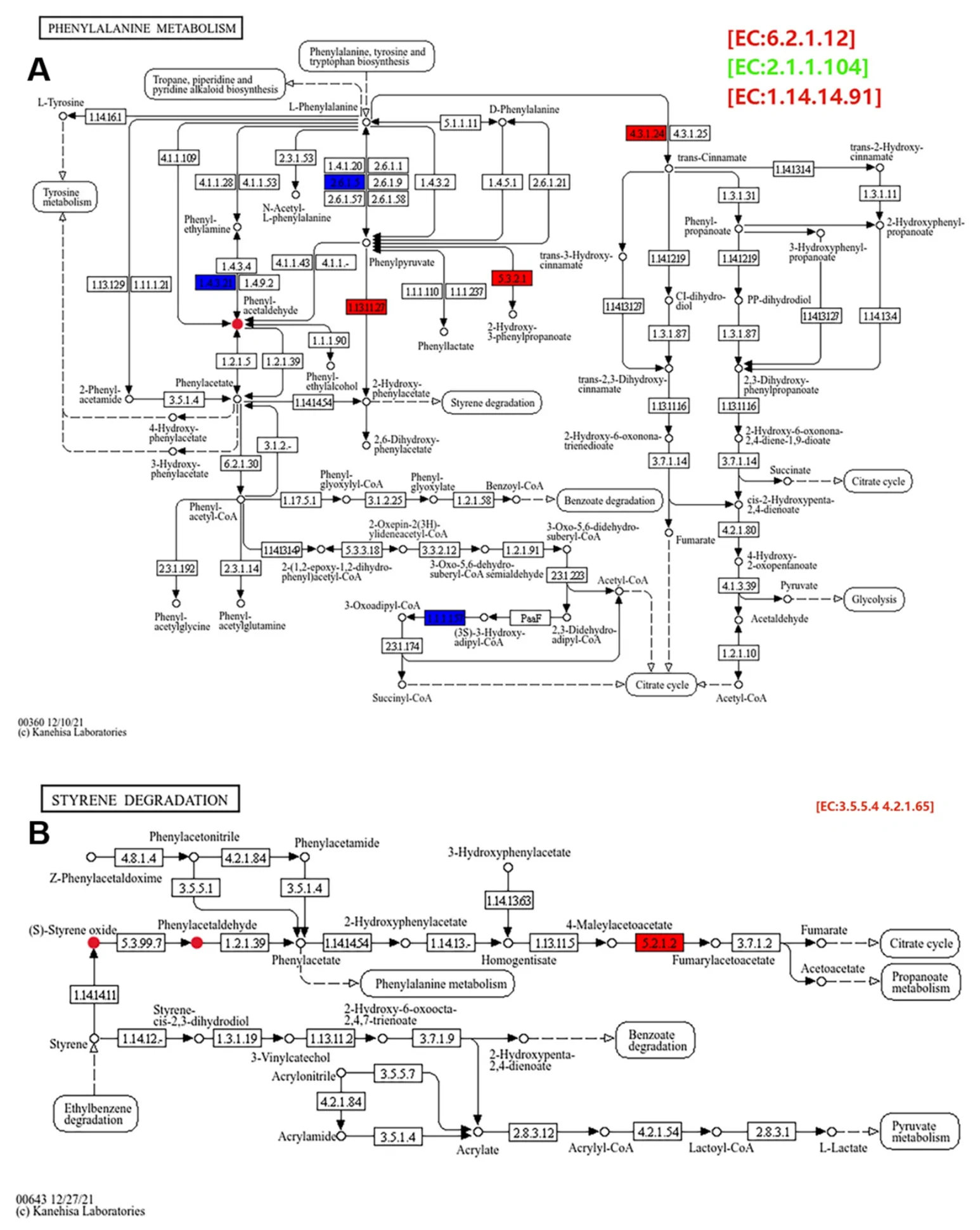
Metabolite–gene KEGG pathway analysis: (**A**) phenylalanine metabolism pathway map; (**B**) styrene degradation pathway map. In the diagrams, circular nodes represent metabolites, while rectangular boxes denote enzymes encoded by the corresponding genes. Red coloration indicates upregulation, green coloration indicates downregulation, and blue coloration signifies that the genes encoding the respective enzymes exhibit both upregulated and downregulated expression. The EC numbers added in the upper right corner of the figure represent enzymes that are enriched in this metabolic pathway but are not displayed in the pathway diagram.

The results of the integrated KEGG pathway analysis for differential metabolites and genes are displayed in Figure 6. Figure 6A illustrates the phenylalanine metabolism pathway, where phenylacetaldehyde was identified as a significantly upregulated metabolite. The pathway map indicates that during *B. variegata* anthesis, the predominant metabolic flow within this pathway is directed toward phenylacetaldehyde. The majority of genes encoding copper amine oxidase [EC:1.4.3.21], which mediates the interconversion between phenylacetaldehyde and phenylethylamine, were downregulated, suggesting attenuated flux between these two metabolites. Furthermore, all genes encoding phenylalanine/histidine ammonia-lyase [EC:4.3.1.24], which catalyzes the formation of trans-cinnamate, were upregulated, indicating that trans-cinnamate and its downstream metabolites represent an important branch of metabolic flow. Additionally, the upregulation of 4-hydroxyphenylpyruvate dioxygenase [EC:1.13.11.27] directs metabolic flux toward the styrene degradation pathway. Figure 6B depicts the styrene degradation pathway, which is interconnected with phenylalanine metabolism. Significantly upregulated metabolites in this pathway included phenylacetaldehyde and (S)-styrene oxide, accompanied by upregulation of a gene encoding glutathione S-transferase [EC:5.2.1.2]. The pathway map indicates that the principal fate of the accumulated phenylacetaldehyde is conversion to phenylacetate, feeding back into phenylalanine metabolism, or diversion toward pathways such as the citrate cycle (TCA cycle) via the action of glutathione S-transferase.

Pathway maps for biosynthesis of terpenoids and steroids and degradation of aromatic compounds are provided in Figures S5 and S6, respectively; however, the differential metabolites and genes enriched in these pathways are not explicitly represented within the provided pathway diagrams.

### 3.7. Quantitative Changes of Key Floral Aroma Components Between Floral Buds and Flowers

**Figure 7:**
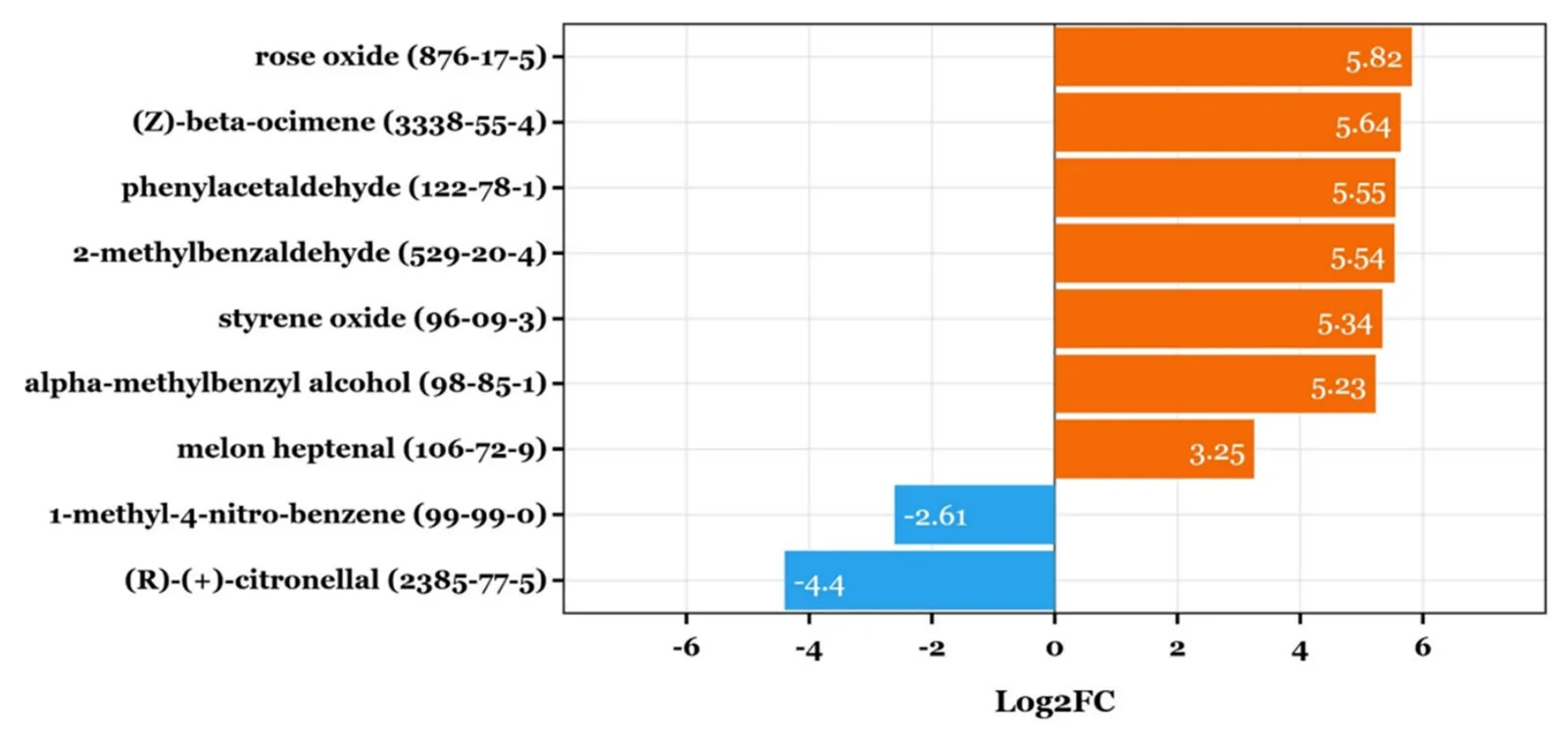
Differences in the content of key volatile metabolites between flowers and floral buds. The vertical axis indicates the log_2_(fold change) in compound content between flowers and floral buds, where values above zero indicate higher content in flowers (orange), values below zero indicate higher content in floral buds (blue), and the further from zero, the greater the difference.

Figure 7 illustrates the differences in the content of key floral scent components between flowers and floral buds. (R)-(+)-citronellal and 1-methyl-4-nitro-benzene were the two volatiles that showed significant downregulation in flowers, with reductions of approximately 21-fold and 6-fold, respectively. In contrast, the key floral aroma components of *B. variegata*—which are primarily responsible for the floral, sweet, and fruity notes—were markedly upregulated in flowers. The specific fold-changes (Flower/Bud) for these major constituents were as follows: rose oxide increased by about 56-fold, (Z)-β-ocimene by about 50-fold, phenylacetaldehyde by about 47-fold, 2-methylbenzaldehyde by about 47-fold, styrene oxide by about 41-fold, alpha-methylbenzyl alcohol by about 37-fold, and melon heptenal by about 10-fold.

## 4. Discussion

### 4.1. B. variegata-Specific Floral Scent Profile and Key Aroma Components

Floral volatile organic compound (VOC) composition varies considerably among plant species, giving rise to distinct, species-specific floral scent profiles. For instance, key aroma components in rose include phenethyl alcohol, citronellol, heneicosane, pentadecane, eugenol, methyleugenol, and geraniol [28,29,30]. In tulip, principal constituents comprise eucalyptol, d-limonene, linalool, trans-β-ocimene, α-pinene, α-farnesene, caryophyllene, geranyl acetone, β-ionone, benzaldehyde, acetophenone, 3,5-dimethoxytoluene, benzyl alcohol, methyl salicylate, 2-phenylethanol, decanal, cis-3-hexenol, cis-3-hexenyl acetate, 2-hexenal, and octanal [12]. In peony, phenylethyl alcohol, β-caryophyllene, linalool, nerol, and (R)-citronellol are among the key contributors [31]. In lavender, linalool, linalyl acetate, 1,8-cineole, and α-terpineol represent the primary aroma-active compounds [32,33].

In the present study, the key aroma components of *B. variegata* were identified and primarily include rose oxide, (Z)-β-ocimene, phenylacetaldehyde, 2-methylbenzaldehyde, styrene oxide, α-methylbenzyl alcohol, 4-phenyl-2-butanol, β-ocimene, 2-isobutyl pyrazine, and melon heptenal. The floral scent profile corresponding to these volatiles is predominantly characterized by fruity, sweet, floral, green, woody, herbal, citrus, phenol, fresh, and spicy odor notes.

### 4.2. Changes in Floral Volatile Organic Compounds Between Floral Buds and Flowers of B. variegata and Their Ecological Functions

Floral volatile organic compounds serve as crucial signals mediating interactions between plants and environmental factors, particularly pollinators [11,12]. During the transition from floral bud to flower, the floral volatiles of *B. variegata* undergo pronounced alterations. (R)-(+)-Citronellal, one of the volatiles unique to floral buds, exhibited a marked downregulation upon anthesis. Citronellal has been reported to possess insect repellent and antimicrobial activities [34,35], implying a significant role in the defense of *Bauhinia* floral buds against herbivores and pathogens. The concomitant downregulation of 1-methyl-4-nitro-benzene, a volatile characterized by a bitter taste, suggests its potential involvement in a bud defense strategy analogous to that of citronellal. This transition from a predominantly defense-oriented mode at the bud stage to an attraction-oriented mode at anthesis represents a precisely regulated adaptation to pollinator activity. In *Antirrhinum majus*, the biosynthesis and emission of methyl benzoate are developmentally regulated [36]. Similarly, in *Petunia hybrida*, the expression of multiple genes associated with floral scent volatile biosynthesis is synchronously upregulated during flower opening [37,38].

Floral scent volatiles play an indispensable role in plant flowering and reproduction. Phenylacetaldehyde constitutes a prominent floral aroma component in numerous plant species [39,40], including *B. variegata*, where its abundance in flowers was 47-fold higher than in buds, representing a substantial upregulation. Phenylacetaldehyde also functions as a critical olfactory signal for pollinator attraction, exhibiting strong attractiveness to moths and other insects [41]. Additionally, it is recognized as a principal contributor to tea aroma [39]. Rose oxide, renowned for its intense rosy fragrance, is a key constituent of rose flowers and essential oil-derived fragrance products [42,43]. β-Ocimene is likewise a well-established contributor to aroma and a pollinator attractant, representing a significant component of floral scent in diverse plant taxa [41,44]. Melon heptenal is also a recognized aroma compound with widespread applications in the fragrance industry [45].

### 4.3. Metabolic Changes and Transcriptional Regulation During Anthesis in B. variegata

To elucidate the molecular mechanisms underlying changes in aroma composition during *B. variegata* anthesis, we integrated metabolomic and transcriptomic data to analyze key metabolic pathways. This analysis revealed that expression alterations in several key enzyme-encoding genes are intimately associated with flowering.

Within the phenylalanine metabolism pathway, all three detected *B. variegata* phenylalanine/histidine ammonia-lyase genes were upregulated in flowers. Phenylalanine ammonia-lyase (PAL) is a pivotal enzyme in phenylalanine metabolism, channeling carbon flux from primary metabolites into phenylpropanoid-derived secondary metabolites, which are crucial for plant growth, development, and environmental adaptation [46]. PAL isoforms are functionally diverse and contribute to various aspects of plant biology. For example, enhanced PAL expression and activity can induce flowering in *Pharbitis* [47]. Phenylalanine/tyrosine ammonia-lyase participates in anthocyanin biosynthesis in *Solanum melongena* [48]. In *Nicotiana attenuata*, phenylalanine ammonia-lyase 4 is involved in the biosynthesis of the floral volatile benzyl acetone, thereby contributing to pollinator attraction [49]. Furthermore, PAL has been implicated in plant resistance to herbivores and pathogens [50,51]. It is therefore plausible that phenylalanine/histidine ammonia-lyase in *B. variegata* plays a critical role during anthesis, although its precise function warrants further validation.

Of the six *B. variegata* copper amine oxidase genes detected within the phenylalanine metabolism pathway, five were downregulated in flowers. Copper amine oxidases participate in alkaloid biosynthesis and, consequently, in plant defense mechanisms [52,53]. The predominant downregulation of copper amine oxidase genes during the transition from bud to flower supports the hypothesis of a developmental shift from defense to attraction in *B. variegata*. Additionally, two *B. variegata* O-methyltransferase genes identified in this pathway were downregulated in flowers. O-Methyltransferases exhibit a close association with plant flowering, potentially regulating floral transition and the expression of scent-related genes through epigenetic mechanisms such as DNA methylation [54,55]. Our results provide additional data supporting a regulatory role for O-methyltransferases in flowering. In contrast, all five *B. variegata* Acyl-CoA synthetase genes detected in the phenylalanine metabolism pathway were upregulated in flowers. Acyl-CoA synthetases are essential for pollen development and are recognized as important regulators of flowering [56,57,58,59].

Within the biosynthesis of terpenoids and steroids pathway, all three detected *B. variegata* squalene synthetase genes were upregulated in flowers. Upregulation of squalene synthetase promotes squalene biosynthesis; squalene can be subsequently converted into sterols and, ultimately, into brassinosteroids, which are phytohormones known to promote plant growth and flowering [60,61].

In the degradation of aromatic compounds pathway, all four detected *B. variegata* aldo–keto reductase genes were downregulated in flowers. Aldo–keto reductases catalyze the reduction of aldehydes to their corresponding alcohols. Their downregulation likely favors the accumulation of aroma-active aldehydes such as phenylacetaldehyde, representing a strategic mechanism by which the plant conserves scent molecules during the flowering phase [62].

## 5. Conclusions

Through integrated metabolomic and transcriptomic analyses, this study provides the first systematic elucidation of the flower volatile organic compound (VOC) composition, floral scent profile characteristics, key aroma constituents, and the molecular mechanisms underlying changes in floral volatiles during anthesis in *B. variegata*. A total of 1214 volatile compounds were identified across floral buds and flowers, encompassing 239 odor-active compounds and 35 differential odor-active compounds. Flavor statistics revealed that the floral scent of *B. variegata* is predominantly characterized by fruity, sweet, floral, green, woody, herbal, citrus, phenol, fresh, and spicy. Compared to floral buds, the vast majority of differential odor-active compounds were markedly upregulated in fully opened flowers, notably including key floral aroma constituents such as phenylacetaldehyde, rose oxide, (Z)-β-ocimene, 2-methylbenzaldehyde, and melon heptenal. Conversely, (R)-(+)-citronellal, a compound with documented defensive functions, and the bitter-tasting substance 1-methyl-4-nitro-benzene were significantly downregulated in flowers, reflecting an ecological strategy shift in *B. variegata* from a defense-oriented mode at the bud stage to a pollinator-attraction mode at anthesis. The upregulation of genes encoding phenylalanine/histidine ammonia-lyase, acyl-CoA synthetase, and squalene synthetase in flowers, coupled with the downregulation of genes encoding copper amine oxidase, O-methyltransferase, and aldo–keto reductase, synergistically facilitated the accumulation of floral aroma compounds such as phenylacetaldehyde and promoted the floral transition. This study provides important data support for a deeper understanding of the ecological interactions between *B. variegata* floral scent and its pollinators, as well as the molecular mechanisms governing floral scent formation. Furthermore, it contributes to the application of *B. variegata* in landscaping, edible flower utilization, and fragrance development.

## Figures and Tables

**Table 1:** Classification, count, and proportion of total flower VOCs.

Classification	Count	Percent
Terpenoids	300	24.71%
Esters	221	18.20%
Ketone	119	9.80%
Heterocyclic compounds	115	9.47%
Alcohols	95	7.83%
Hydrocarbons	74	6.10%
Phenols	58	4.78%
Acids	55	4.53%
Aldehydes	51	4.20%
Aromatics	45	3.71%
Amines	28	2.31%
Ethers	26	2.14%
Nitrogen compounds	15	1.24%
Sulfur compounds	12	0.99%

**Table 2:** Classification and count of differential odor-active compounds.

Classification (Count)	Compounds
Ethers (1)	4-(methylthio)-butanenitrile
Ketone (1)	2-octanone
Nitrogen compounds (1)	1-methyl-4-nitro-benzene
Amines (2)	2,4,6-trimethyl-benzenamine; o-toluidine
Aromatics (2)	1,2,3,4-tetramethyl benzene; butylbenzene
Alcohols (3)	4-phenyl-2-butanol; 5-methyl-2-furanmethanethiol; alpha-methylbenzyl alcohol
Aldehydes (3)	benzeneacetaldehyde; melon heptenal; 2-methylbenzaldehyde
Phenols (4)	2-ethylphenol; 3-ethylphenol; 4-ethylphenol; 2-methylphenol
Esters (5)	3-methylbutyl pentanoate; methyl 2-octynoate; methyl benzoate; hexyl octanoate; 1-octenyl-3-propanoate
Terpenoids (6)	(Z)-β-ocimene; β-ocimene; 4-isopropylbenzaldehyde; (R)-(+)-citronellal; (2R,4S)-rose oxide; (2S,4R)-rose oxide
Heterocyclic compounds (7)	styrene oxide; 2-isobutyl pyrazine; 2-pentylfuran; 2-ethyl-5-methyl-pyrazine; 2-methoxy-3,5-dimethylpyrazine; 2-methyl-3-isopropylpyrazine; 2-ethyl-6-methyl-pyrazine

**Table 3:** Seven metabolites annotated to KEGG pathways.

CAS	Compounds	Class	Formula
96-09-3	styrene oxide	Heterocyclic compound	C_8_H_8_O
529-20-4	2-methylbenzaldehyde	Aldehyde	C_8_H_8_O
122-78-1	phenylacetaldehyde	Aldehyde	C_8_H_8_O
95-48-7	2-methylphenol	Phenol	C_7_H_8_O
2385-77-5	(R)-(+)-citronellal	Terpenoids	C_10_H_18_O
122-03-2	4-isopropylbenzaldehyde	Terpenoids	C_10_H_12_O
123-07-9	4-ethylphenol	Phenol	C_8_H_10_O

**Table 4:** Statistical summary of KEGG pathway classification for differential odor-active compounds.

Kegg Pathway	Ko_ID	Count	IndexList	CIDList
Styrene degradation	ko00643	2	96-09-3; 122-78-1	C20782; C00601
Metabolic pathways	ko01100	5	96-09-3; 529-20-4; 122-78-1; 95-48-7; 122-03-2	C20782; C07214; C00601; C01542; C06577
Microbial metabolism in diverse environments	ko01120	7	96-09-3; 529-20-4; 122-78-1; 95-48-7; 2385-77-5; 122-03-2; 123-07-9	C20782; C07214; C00601; C01542; C09848; C06577; C13637
Xylene degradation	ko00622	2	529-20-4; 122-03-2	C07214; C06577
Degradation of aromatic compounds	ko01220	3	529-20-4; 95-48-7; 122-03-2	C07214; C01542; C06577
Phenylalanine metabolism	ko00360	1	122-78-1	C00601
Toluene degradation	ko00623	1	95-48-7	C01542
Pinene, camphor and geraniol degradation	ko00907	1	2385-77-5	C09848
Biosynthesis of terpenoids and steroids	ko01062	1	2385-77-5	C09848
Biosynthesis of secondary metabolites	ko01110	1	2385-77-5	C09848
Bisphenol degradation	ko00363	1	123-07-9	C13637

## Data Availability

All data are provided in the Supplementary Materials; further inquiries can be directed to the corresponding author.

## Supplementary Materials

The following supporting information can be downloaded at https://www.mdpi.com/article/10.3390/metabo16090611/s1, Figure S1: Inflorescence of *Bauhinia variegata*. Figure S2: Floral scent profile of *Bauhinia variegata* floral buds. Figure S3: Floral scent profile of *Bauhinia variegata* flowers. Figure S4: Venn diagram of odor-active compounds in floral buds and flowers of *Bauhinia variegata*. Figure S5: Biosynthesis of terpenoids and steroids pathway. Figure S6: Degradation of aromatic compounds pathway. Table S1: Relative odor activity values of all detected volatile metabolites. Table S2: Odor-active metabolites in floral buds. Table S3: Odor-active metabolites in flowers. Table S4: Special metabolites in floral buds or flowers. Table S5: Significantly differential odor-active metabolites. Table S6: Differential odor-active metabolites annotated with KEGG compound and pathway IDs. Table S7: Significantly differentially expressed genes in the relevant KEGG pathways.
